# Whole Exome Sequencing Reveals Mutations in Known Retinal Disease Genes in 33 out of 68 Israeli Families with Inherited Retinopathies

**DOI:** 10.1038/srep13187

**Published:** 2015-08-26

**Authors:** Avigail Beryozkin, Elia Shevah, Adva Kimchi, Liliana Mizrahi-Meissonnier, Samer Khateb, Rinki Ratnapriya, Csilla H. Lazar, Anat Blumenfeld, Tamar Ben-Yosef, Yitzhak Hemo, Jacob Pe’er, Eduard Averbuch, Michal Sagi, Alexis Boleda, Linn Gieser, Abraham Zlotogorski, Tzipora Falik-Zaccai, Ola Alimi-Kasem, Samuel G. Jacobson, Itay Chowers, Anand Swaroop, Eyal Banin, Dror Sharon

**Affiliations:** 1 Departments of Ophthalmology, Hadassah-Hebrew University Medical Center, Jerusalem, Israel; 2 Neurobiology-Neurodegeneration & Repair Laboratory, National Eye Institute, National Institutes of Health, Bethesda, MD 20892, USA; 3 Molecular Biology Center, Interdisciplinary Research Institute on Bio-Nano Sciences, Babes-Bolyai-University, Cluj-Napoca, 400271, Romania; 4 Rappaport Faculty of Medicine, Technion, Haifa, Israel; 5 Human Genetics, Hadassah-Hebrew University Medical Center, Jerusalem, Israel; 6 Dermatology, Hadassah-Hebrew University Medical Center, Jerusalem, Israel; 7 Institute of Human Genetics, The Galilee Medical Center, Naharia, Israel; The Galilee Faculty of Medicine, Bar-Ilan, Israel; 8 Ha’emek hospital, Afula, Israel; 9 Department of Ophthalmology, Scheie Eye Institute, University of Pennsylvania, Philadelphia, Pennsylvania, USA

## Abstract

Whole exome sequencing (WES) is a powerful technique for identifying sequence changes in the human genome. The goal of this study was to delineate the genetic defects in patients with inherited retinal diseases (IRDs) using WES. WES was performed on 90 patient DNA samples from 68 families and 226 known genes for IRDs were analyzed. Sanger sequencing was used to validate potential pathogenic variants that were also subjected to segregation analysis in families. Thirty-three causative mutations (19 novel and 14 known) in 25 genes were identified in 33 of the 68 families. The vast majority of mutations (30 out of 33) have not been reported in the Israeli and the Palestinian populations. Nine out of the 33 mutations were detected in additional families from the same ethnic population, suggesting a founder effect. In two families, identified phenotypes were different from the previously reported clinical findings associated with the causative gene. This is the largest genetic analysis of IRDs in the Israeli and Palestinian populations to date. We also demonstrate that WES is a powerful tool for rapid analysis of known disease genes in large patient cohorts.

Inherited retinal diseases (IRDs) are visual disorders that can be congenital [e.g., LCA (Leber congenital amaurosis)] or appear later in life [e.g., RP (retinitis pigmentosa)]. LCA is a severe nonsyndromic retinal dystrophy, with a prevalence of 1:80,000 in the USA. It is characterized by severe visual impairment at birth, a non-detectable electroretinogram (ERG), nystagmus, hypermetropia, sluggish or absent pupillary responses, and oculodigital reflexes^1^,^2^. LCA is genetically heterogeneous with at least 19 identified genes that are associated with photoreceptor development and/or function^2^. In contrast, RP is generally relatively less severe and more clinically heterogeneous with a later age of onset, yet with a higher prevalence (1:4500 in Europe and USA)^3^,^4^,^5^,^6^. The prevalence of nonsyndromic RP in the vicinity of Jerusalem is estimated at about 1:2100^7^. RP is characterized by night blindness, followed by a gradual loss of peripheral vision, a progressive degeneration of photoreceptors which eventually leads to visual impairment of a variable severity that might end with complete blindness^8^. Patients with RP may have severely reduced or total absence of a-waves on ERG testing and bone spicule-like pigmentations (BSPs), attenuation of retinal vessels and a waxy pallor of the optic disc on funduscopy^8^,^9^.

Molecular studies of IRDs have yielded mutations in over 200 causative genes so far. The large number of identified genes for retinal dystrophies makes it difficult and expensive to screen all genes systematically in a cohort of patients. Next generation sequencing (NGS) is an efficient tool for high throughput analysis of genomic DNA and transcriptomes. One of the NGS applications is whole exome sequencing (WES) that has been used to detect mutations in coding regions. WES requires a relatively low sequencing volume and the process is rapid and easier compared to Sanger sequencing for the analysis of a large set of candidate genes and patient cohorts. In addition, WES requires less computational resources and is cheaper than whole genome sequencing (WGS). During the last few years, WES has been successfully used to identify genetic causes of IRDs^10^,^11^,^12^,^13^,^14^,^15^,^16^.

The Israeli and Palestinian populations are characterized by a relatively high number of consanguineous/intracommunity marriages. These features result in long homozygous genomic regions, complicating the identification of disease-causing mutations^17^. Recently, marriage patterns seem to be changing among Jews, with enhanced rate of marriages between different Jewish communities^18^, thereby leading to an increased proportion of compound heterozygous mutations, which are even more challenging to identify using the traditional homozygosity mapping approach.

Here, we report WES analysis of 90 exomes in 68 families leading to the identification of 33 mutations in Israeli and Palestinian patients/families in genes that have previously been associated with retinal dystrophies.

## Results

**Figure 1:**
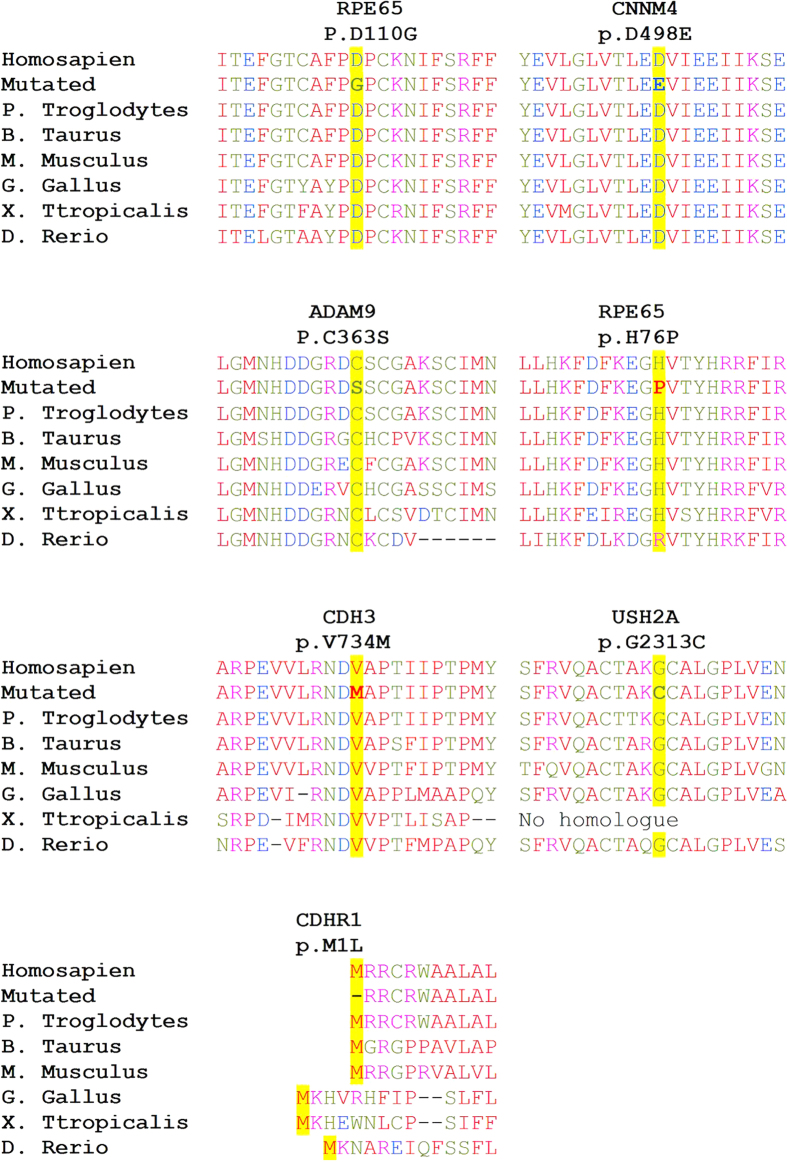
Amino acid alignments around seven novel missense mutations. The altered residues (marked in yellow) are fully conserved through all species in almost all cases. The aa type is color-coded: small aa in red, acidic in blue, basic in magenta, and hydroxyl + amine + basic in green.

Ninety patients from 68 Israeli and Palestinian families who were diagnosed with autosomal dominant (AD) or autosomal recessive (AR) retinal dystrophy [primarily with RP (75 patients) and LCA (10 patients)] were recruited for this study. Whole genome single nucleotide polymorphism (SNP) array analysis was performed on DNA samples from 19 patients revealing a large number of homozygous regions including many candidate genes. Subsequently, WES analysis was performed on all 90 samples using two different platforms (see Methods section). Data analysis of 226 known IRD-causing genes revealed the identification of 33 causative mutations in 25 genes in 33 of the families (49% - see Tables 1 and 2 and Supplementary Figure S1 online). The vast majority of mutations (91% - 30 out of 33) have not been reported thus far in the Israeli and Palestinian populations. Nineteen of the 33 identified mutations (58%) were novel: 6 nonsense, 6 frameshift and 7 missense mutations. Amino-acid sequence alignment of the relevant protein regions demonstrated conservation of mutant allele in all studied cases (Fig. 1). In addition, these missense mutations are likely pathogenic as suggested by three prediction programs and calculated minor allele frequency (MAF) values from various databases (Table 3). Based on the above-mentioned analyses, the mutations were divided into definite pathogenic mutations (Table 1) and possible pathogenic mutations (Table 2).

Due to the unique structure of the populations we are studying, a substantial percentage of sequence changes appear to be founder mutations and are present in multiple families who share the same ethnicity. We therefore screened each of the identified mutations in a set of ethnicity-matched patients with a similar retinal phenotype. Our analysis revealed that 9 out of the 33 mutations (27%) appear in multiple families (Table 1). Interestingly, a single *CNGB1* mutation (c.2284C>T) was detected in a homozygous state in 4 out of 8 RP families of Bukharian Jewish origin.

In nine families, homozygosity mapping data was available prior to WES analysis. The average size and rank of the homozygous region harboring the genetic defect was 22.5 Mb and 4.3, respectively (Tables 1 and 2). However, homozygosity mapping *per se* was not efficient enough for gene identification in these cases. In some of the families we identified unique inheritance patterns or clinical features as detailed below.

### Co-occurrence of two different inherited retinal diseases in the same family

Two of the families recruited for the study, MOL0367 and MOL1145, are of Druze origin, and each contains two family branches with different retinal diseases as demonstrated by the genetic analysis as well.

**Figure 2:**
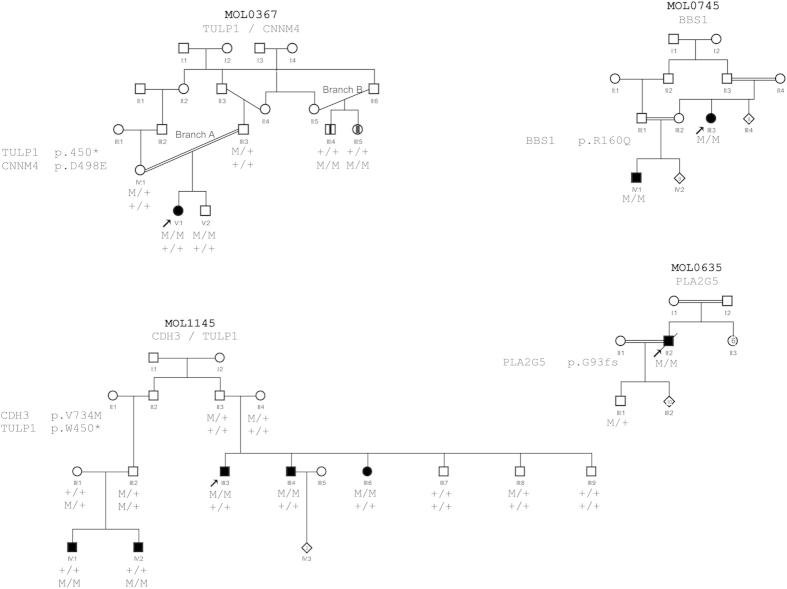
Pedigrees of 4 families discussed in details in the paper. The family number and the mutated gene/s are noted above each pedigree. The genotype for each mutation is listed below each individual’s symbol. Consanguinity is marked by double lines.

In family MOL0367, three family members were clinically diagnosed with retinal degeneration (Table 4 and Fig. 2): an isolate case with LCA and Coats disease (V:1- branch A) and two siblings initially diagnosed with RP (III:4 and III:5- Branch B). We previously reported the results of homozygosity mapping analysis that revealed a *TULP1* homozygous nonsense mutation (c.1349G>A, p.W450*) which cosegregated with LCA in branch A of the family^17^. None of the family members in branch B, however, carried this mutation. HM analysis of the two siblings in branch B revealed five large homozygous regions, none harbored a known gene for nonsyndromic IRD. We subsequently performed WES analysis on the DNA sample of III:4 and identified a novel missense sequence variant (c.1494C>A, p.D498E) in the *CNNM4* gene which is located in the largest shared homozygous region. The c.1494C>A sequence change was absent in WES databases (see Methods section), is highly conserved during evolution (Fig. 1), and suggested to be damaging by the prediction programs. Following the genetic findings, patient III:4 reported dysmorphic teeth and amelogenesis imperfecta. The phenotype of these siblings was therefore revised to Jalili syndrome.

**Figure 3:**
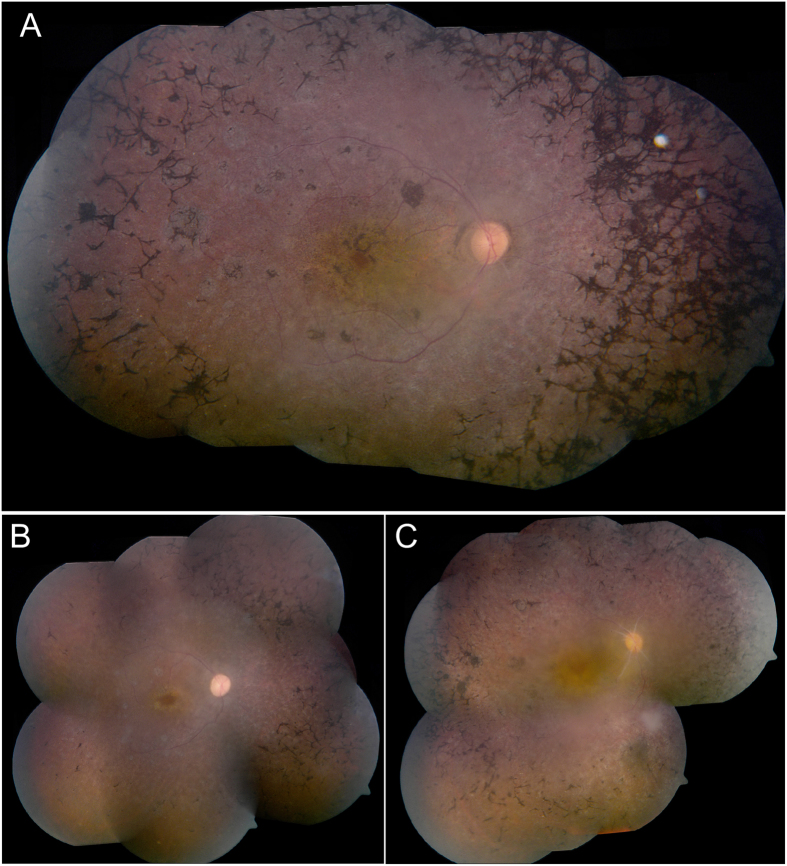
Fundus phenotype of three affected individuals of MOL1145 with a homozygous *CDH3* mutation. (**A**) Fundus appearance of individual III:3. At the age of 34 years, he demonstrates narrowed blood vessels, waxy pallor of the optic nerve, bone-spicule like pigmentations, typical for RP, and severe macular atrophy. His brother III:4 (**B**) and sister III:6 (**C**) at the age of 30 and 33 respectively, demonstrated the same fundus appearance, but with less bone spicule-like pigmentations.

Following the identification of the p.W450* nonsense mutation in MOL0367, we screened the mutation in a set of 33 families of Druze origin. We identified the p.W450* mutation in one of these families, MOL1145, which consists of two different branches with affected family members. Two brothers (IV:1 and IV:2) who were diagnosed with LCA/early onset RP, were found to be homozygous for the p.W450* mutation. Three siblings (III:3, III:4, III:6- Fig. 2) who belong to a different branch of the family and were diagnosed with RP, were negative for the *TULP1* mutation. We subsequently performed WES analysis on the Index case and identified a novel homozygous missense change (p.V734M) in the *CDH3* gene. The p.V734M change was absent in WES databases, fully cosegregated in this family branch, is highly conserved during evolution (Fig. 1), and is predicted by two of the three prediction online programs as damaging. We screened a set of ethnicity-matched controls and identified the mutation in a heterozygous state in 4 out of 94 healthy controls from the same village, with a population size of 4300 individuals who are mostly related to each other. Mutations in *CDH3* have been reported previously to cause congenital hypotrichosis with juvenile macular dystrophy^19^,^20^,^21^. A thorough examination by a dermatologist did not reveal any skin or hair abnormalities. The 3 siblings were 30–34 yrs of age at the time of the examination. All had a visual acuity of hand movement at a distance of 5–45 cm, with no detectable ERG response (cone, rod and mixed responses- Table 4), and fundus examination revealed typical and severe features of RP, including narrowed blood vessels, waxy pallor of the optic nerve, bone-spicule like pigmentation as well as macular atrophy (Fig. 3).

### A BBS1 mutation in patients with nonsyndromic RP

Family MOL0745 contains two relatives who were diagnosed with nonsyndromic RP (Table 4) and their samples underwent SNP array analysis, revealing only one shared homozygous region of 8 Mb on chromosome 11 with no obvious candidates. Screening of other known disease-causing mutations in this origin was negative. The DNA samples of the two patients underwent WES analysis, and a homozygous sequence change in the *BBS1* gene (known to cause mainly Bardet Biedl syndrome) was identified in the largest homozygous region. This variant, c.479G>A, affects the last nucleotide of exon 5 and was previously shown to partially affect BBS1 splicing^22^.

### A *PLA2G5* frameshift mutation in a patient with late-onset RP

The index case of the consanguineous family MOL0635 had good vision until the age of 85 years and was diagnosed with late-onset RP at 90. We performed SNP array on 2 family members and WES on the index case, leading to the identification of a homozygous frameshift mutation in the *PLA2G5* gene, which is located in the largest homozygous region. Two *PLA2G5* mutations were reported previously to cause a mild retinal phenotype, termed benign fleck retina, with no retinal dystrophy^23^. The patients reported previously were much younger than the patient we describe here, and the two phenotypes might represent different stages of the same disease.

## Discussion

Genetic heterogeneity and phenotypic variability of IRDs limit our ability to efficiently identify the genetic cause of disease using a candidate gene approach, and therefore we have restricted options to provide accurate genetic counseling and gene-based therapies (e.g. gene augmentation therapy)^24^. Many tools have been developed to make this process more efficient (including mutation detection arrays and homozygosity mapping), but each tool has significant limitations and is efficient only in specific populations and family structures. WES allows us to examine a large number of nucleotide changes in coding exons simultaneously and compare these to other family members or individuals of the same ethnic origin.

In the current study, we have identified causative mutations in 25 genes in 33 out of 68 studied families (49%). This is in-line with the recent data reported in cohorts of patients from Saudi-Arabia (81 out of 149 families- 54%)^25^, China (79 out of 157 families- 50%)^26^, Thailand (11 out of 20 families- 55%)^27^, and Northern Ireland (49 out of 82 families- 60%)^28^. The fact that a mutation can be identified in only about half of the cases, further highlights the genetic complexity of retinal degenerative diseases. Although one can assume that some mutations in known genes are missed by WES analysis (e.g., intronic changes that affect mRNA splicing, 5′ and 3′ UTR changes affecting mRNA production and stability, and large deletions/insertions of one or more full exons), it is reasonable to predict that additional retinopathy genes remain to be identified. In addition, since the number of variants obtained by WES analysis is large, the causing-mutation might be over-looked in a few cases due to the complex data analysis process.

The efficiency of WES as a gene identification tool can be compared to autozygosity mapping that was shown previously to yield positive results in 13%^29^ and 12%^30^ of families in the European population, and 13% (16 out of 125 families) reported by us in the same studied population^17^. In the current study, SNP array data were available for 17 of the 62 families, but the homozygosity mapping analysis failed to reveal the causative mutation. The subsequent WES analysis led to the identification of causative mutations in 11 families. Interestingly, the average size of the homozygous region harboring the causative mutation was smaller in this group of 11 families compared to families in whom the causative mutation was identified by homozygosity mapping (22.5 Mb comparing to 26.7 Mb)^17^. Similarly, the rank of the region was higher in the WES group (4.3 comparing to 2). This can be explained by the relatively large number of homozygous regions that exist in the genomes of patients from our populations^17^, thereby complicating homozygosity mapping analysis and limiting the analyses to the largest regions only. WES, on the other hand, allows one to analyze all regions at once. In addition, WES analysis also assisted in the identification of compound heterozygous mutations in a family that was found to be negative in the homozygosity mapping analysis (family MOL0927). These results suggest that WES is a relatively powerful tool for mutation identification.

**Figure 4:**
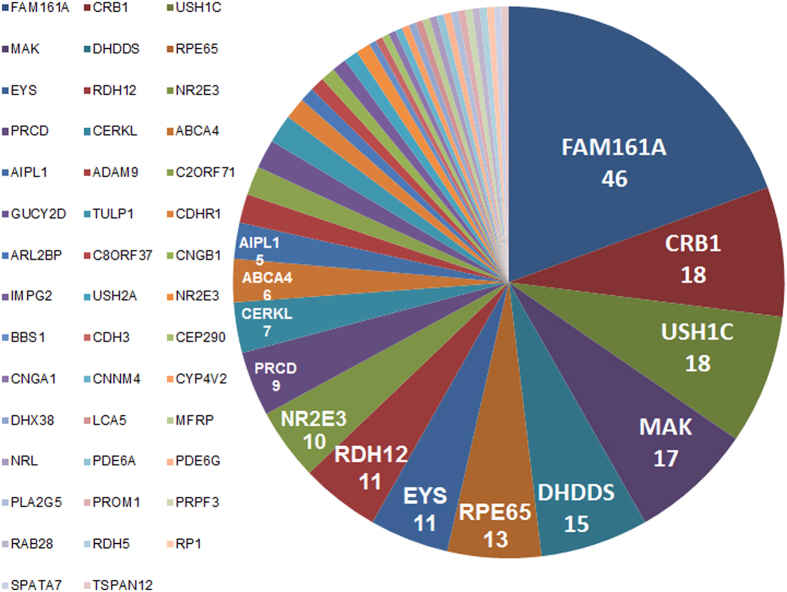
Genetic architecture of autosomal recessive IRDs in the Israeli and Palestinian populations. The number of families with mutations in the related gene is indicated below the gene symbol.

Thus far, mutations in 30 genes have been associated with retinal dystrophies in 174 Israeli and Palestinian families (Supplementary Table S3 online). We now report mutations in 12 additional genes (in a total of 71 additional families); these genes were not previously associated with retinopathies in the Israeli and Palestinian populations, thus greatly expanding the current list of genes by 40%. The genes in which mutations were identified are listed in Fig. 4 and include both relatively frequent mutations (e.g., in the *FAM161A, CRB1, USH1C, MAK*, and *DHDDS* genes) as well as a large number of genes that are responsible for the disease in only a single family. The existence of a frequent founder mutation allows one to increase the efficiency of mutation detection by pre-screening for frequent mutations that are present in the relevant sub-population. WES can then be performed only on the remaining families.

We also present data of two families with multiple causes of IRDs: MOL0367 with *TULP1* and *CNNM4* mutations and MOL1145 with *TULP1* and *CDH3* mutations, both of Druze origin. The *CDH3*-related phenotype we report here (RP) is different from that reported in the literature, congenital hypotrichosis with juvenile macular dystrophy^20^,^21^,^31^,^32^,^33^, which initially involves the macular region, spreads to the periphery, and later on the RPE is also involved^19^,^34^. The three affected siblings of MOL1145 show a unique and uniform phenotype that is compatible with RP with macular involvement and no skin abnormalities. Although the *CDH3* variant is prevalent among Druze of the same village (4 carriers out of 94 individuals), previous studies showed that disease-causing mutations can be highly prevalent in such closed populations^35^,^36^.

Another feature that complicates gene identification in retinal diseases is the fact that mutations in the same gene can cause either a syndrome or a nonsyndromic phenotype. An excellent example is the set of 14 genes that were reported initially to cause Bardet-Biedl syndrome when mutated. Recent data, mainly using WES analysis, revealed that specific mutations in six of these genes can cause nonsyndromic RP^22^,^37^,^38^,^39^. Interestingly, one of the families we analyzed (MOL0745 of an Arab-Muslim origin) was found to harbor an apparent missense mutation in the *BBS1* gene that was reported previously in a German family to cause a relatively mild splicing defect leading to a nonsyndromic phenotype^22^.

Family MOL0635 presents yet another interesting case from the clinical point of view. The index patient was diagnosed with late-onset RP at the age of 88 and had no previous visual complaints. WES revealed a homozygous *PLA2G5* frameshift mutation. Mutations in this gene were previously reported to cause a relatively mild retinal phenotype- benign fleck retina^23^, which is characterized by lesions on the fundus which appeared as discrete, bright white or yellow flecks. The reported patients were relatively young at ages 6 to 37 years. Since the index patient of family MOL0635 was not examined by an ophthalmologist at an early age and is the only affected individual in his family, one can propose that benign fleck retina is an early stage of the disease and can later on deteriorate to retinal degeneration.

In summary, this is the largest comprehensive genomic research of IRD patients from the Israeli and Palestinian populations to date. In this study, we showed that WES by itself can be very useful in identifying mutations that cause IRD in the Israeli and Palestinian populations, even without prior genetic analysis (e.g. HM). The 12 identified genes, which were not reported previously as causative genes in the Israeli and Palestinian populations, are now new candidates for screening during genetic counseling for RP patients.

## Materials and Methods

### Patients and Clinical evaluation

All methods used in the study were carried out in accordance with the approved guidelines. Ninety patients, belonging to 68 Israeli and Palestinian families, were recruited for this study with the diagnoses of autosomal dominant (AD) or autosomal recessive (AR) retinopathy (including RP, LCA, enhanced s-cone syndrome [ESCS] and familial exudative vitreous retinopathy [FEVR]). All participants in the study signed an informed consent that adhered to the tenets of the declaration of Helsinki before drawing a blood sample for molecular analysis. Ethical approval for this study was obtained from the local Helsinki committee at the Hadassah Medical Center. The ocular diagnosis was determined using a full ophthalmic examination, full-field electroretinography (FFERG), electro-oculography (EOG), color vision testing using the Farnsworth D-15 Panel test and Ishihara test, optical coherence tomography (OCT), color, infrared and fundus autofluorescence (FAF) imaging, and fluorescein angiography (FA), as detailed previously^17^.

### Genetic analysis

Whole genome single nucleotide polymorphism (SNP) arrays were performed on DNA samples of 19 patients (who belong to 17 families) using different SNP microarrays platforms including Affymetrix 10K, 6.0 and Illumina 6K arrays. The data was analyzed using the HomozygosityMapper online program (http://www.homozygositymapper.org/), and homozygous regions were identified. A homozygous region was defined as at least 39 consecutive homozygous SNPs in 10k arrays and at least 3900 consecutive homozygous SNPs in 6.0 arrays. All homozygous regions in each family were searched for genes that were reported previously to cause a retinal disease. WES analysis using NimbleGen V2 (44.1 Mbp) paired-end sample preparation kit and Illumina HiSeq2000 at 31X coverage (at Otogenetics Corporation) was performed on 55 samples. The remaining 35 samples underwent an in-house WES analysis as follows: genomic DNA (3 μg) was fragmented by Covaris and subjected to whole exome capture using Agilent SureSelectXT Target Enrichment Kit (50 Mbp) for Illumina Multiplex Sequencing (Agilent Technologies, Santa Clara, CA), following manufacturer′s instructions. Captured libraries were amplified and converted to clusters using Illumina Cluster Station. Paired-end sequencing was performed on Illumina GAIIx. Approximately 3 GB of sequence was generated per individual, resulting in ~90% coverage of targeted Consensus Coding Sequence project (CCDS) exonic bases, with an average depth of ~80×. Sequence reads were aligned to the human genome reference (UCSC hg 19; http://genome.ucsc.edu/) using the Genomatix Mining Station (GMS) and variants were called and annotated using the DNAnexus software package (https://www.dnanexus.com/). Variant files were annotated using ANNOVAR according to the dbSNP database (built 137) with the following filtering steps: (1) For families with SNP array data, all variants in known IRD genes that are located within homozygous regions were analyzed prior to any other analysis; (2) All variants in 226 known IRD genes (based on RetNet https://sph.uth.edu/retnet/) were analyzed (Supplementary Table S1 online); (3) Variant type: Missense, nonsense, splice-site, stop-loss, insertions and deletions in the coding region were included; (4) Variants that were found in repeat DNA segments were excluded; (5) Variants with minor allele frequency (MAF) greater than 0.5% in the NHLBI Exome Sequencing Project (http://evs.gs.washington.edu/EVS/) (N = 6,500), ExAC server (http://exac.broadinstitute.org/) (N = 66,000) and in an in-house Israeli WES database (N = 408) were excluded; (6) Prediction of the possible effect of each variant was analyzed by 3 prediction online programs [SIFT (http://sift.jcvi.org/), MutationTaster (http://www.mutationtaster.org/) and PolyPhen2 (http://genetics.bwh.harvard.edu/pph2/). The identified sequence variants were genotyped in affected as well as unaffected family members for segregation analysis.

Primers for all the suspected variants were designed using Primer3 online program (http://www.bioinformatics.nl/cgi-bin/primer3plus/primer3plus.cgi/) (see Supplementary Table S2 online). Sanger sequencing of PCR products was performed to verify each mutation, genotype other family members and screen additional patients and ethnicity-matched controls.

## Additional Information

**How to cite this article**: Beryozkin, A. *et al.* Whole Exome Sequencing Reveals Mutations in Known Retinal Disease Genes in 33 out of 68 Israeli Families with Inherited Retinopathies. *Sci. Rep.* **5**, 13187; doi: 10.1038/srep13187 (2015).

## Supplementary Material

Supplementary Information

## Figures and Tables

**Table 1:** A list of definite pathogenic mutations identified in this study.

Gene	DNA change	Proteinchange	MAFbyExAC	Inheritancepattern anddiagnosis*	Origin	Familiesidentifiedby WES	#of additionalfamilies identifiedby targetedmutation screen	Zygosity	Size in Mb of thegene- containinghomozygousregion (rank)
***ADAM9***	**c.1087T>A**	**p.C363S**	**0**	**arRP**	**Iraqi Jew**	**MOL0827 MOL0838**	—	**Homozygous Homozygous**	33.0 (1)**
*AIPL1*	c.211G>T	p.V71F	8*10^−6^	arLCA	Mix Jew	MOL0113	—	Homozygous	
*BBS1*	c.479G>A	p.R160Q	4*10^−5^	arRP	Arab Muslim	MOL0745	—	Homozygous	8.0 (1)**
***CDHR1***	**c.2087_2090delACAA**	**p.D696Afs**	**0**	**arRD**	**Arab Muslim**	**MOL0835**	—	**Homozygous**	
*CNGA1*	c.94C>T	p.R32*	0	arRP	Moroccan Jew	MOL0927	—	Heterozygous	
***CNGA1***	**c.1540C>T**	p.R514*	**0**	**arRP**	**Moroccan Jew**	**MOL0927**	—	**Heterozygous**	
*CNGB1*	c.2284C>T	p.R762C	0	arRP	Buchara Jew	MOL0990	4	Homozygous	
***CNGB1***	**c.2760G>A**	p.W920*	8*10^−**6**^	**arRP**	**Ashkenazi Jew**	**CHRD**	—	**Homozygous**	
***CNNM4***	**c.1494C>A**	**p.D498E**	**0**	**ar Jalili syndrome**	**Druze**	**MOL0367**	—	**Homozygous**	27.8 (1)**
***CYP4V2***	**c.1123delC**	p.L375*	**0**	**arRP**	**Yemenite Jew**	**MOL0816**	**1**	**Homozygous**	3.2 (4)**
*C2ORF71*	c.2950C>T	p.R984*	2*10^−5^	arRP	Jew (Iran, Turkey)	MOL0425	—	Heterozygous	
*C2ORF71*	c.3289C>T	p.Q1097*	4*10^−5^	arRP	Jew (Iran, Turkey)	MOL0425	—	Heterozygous	
*FAM161A*	c.1355_6delCA	p.T452Sfs	4*10^−5^	arRP	Moroccan Jew	MOL0228	26	Homozygous	
*MAK*	c.1284_1285ins353	p.F428fs	0	arRP	Ashkenazi Jew	MOL0826	16	Homozygous	
***NRL***	**c.444_445insGCTGCGGG**	**p.Q148fs**	**0**	**arRP**	**Iraqi Jew**	**MOL1109**	—	**Homozygous**	
*NR2E3*	c.119-2A>C		8*10^−6^	arRP	Arab Muslim	MOL1266	1	Homozygous	
***PDE6A***	**c.1960C>T**	p.Q654*	**0**	**arRP**	**Arab Muslim**	**MOL0271**	—	**Homozygous**	33.4 (5)
*PRPF3*	c.1481C>T	p.T494M	0	adRP	Yemenite Jew	MOL0108	—	Heterozygous	
***PLA2G5***	**c.279_280insG**	**p.G93fs**	**0**	**ar late-onset RP**	**Arab Muslim**	**MOL0635**	—	**Homozygous**	33.6 (3)
***RDH5***	**c.412delA**	**p.M138fs**	**0**	**ar Fundus albipunctatus**	**Arab Muslim**	**MOL1025**	—	**Homozygous**	
*RDH12*	c.295C>A	p.L99I	7*10^−5^	arRP	Moroccan Jew Moroccan Jew Turkish Jew	MOL1042 MOL0589 MOL0279	3	Homozygous Homozygous Homozygous	
*RDH12*	c.377C>T	p.A126V	8*10^−6^	arRP	Arab Muslim	MOL1389	—	Homozygous	
*RPE65*	c.722A>T	p.H241L	0	arRP	Ashkenazi Jew	MOL0942	—	Homozygous	
***RP1***	**c.688G>T**	p.G230*	**0**	**arRP**	**Yemenite Jew**	**MOL0181**	**1**	**Homozygous**	21.4 (5)
***SPATA7***	**c.288T>A**	p.C96*	3*10^−**5**^	**arLCA**	**Arab Muslim**	**MOL0758**	—	**Homozygous**	6 (5)**
*TSPAN12*	c.542G>T	p.C181F	0	arFEVR	Beduin	MOL0513	—	Homozygous	
***TULP1***	**c.852_853insTCCC**	**p.P284fs**	**0**	**arLCA**	**Ethiopian Jew**	**MOL1125**	—	**Homozygous**	
***USH2A***	**c.377delG**	**p.S126fs**	**0**	**arRP**	**Arab Muslim**	**MOL1234**	—	**Heterozygous**	
***USH2A***	**c.6937G>T**	**p.G2313C**	1*10^−**4**^	**arRP**	**Arab Muslim**	**MOL1234**	—	**Heterozygous**	

A sequence variant is considered as a definite pathogenic mutation if one of the following applies: it was previously published as a pathogenic mutation, a novel nonsense, frameshift or splice-site mutation, and a novel missense mutation which is predicted by all prediction programs as damaging. In addition, a variant is considered pathogenic if the variant cosegregated with the disease in the studied family, MAF < 0.001 by ExAC (http://exac.broadinstitute.org/) and was not found in a homozygous state in the ExAC controls. Novel sequence changes are highlighted in Bold.

^*^ar- autosomal recessive; ad- autosomal dominant.

^**^The size of the homozygous region that is shared among two affected family members.

**Table 2:** A list of novel possible pathogenic mutations identified in this study.

Gene	DNA change	Proteinchange	MAF byExAC	Inheritancepattern anddiagnosis*	Origin	Familiesidentifiedby WES	# of additionalfamilies identifiedby targetedmutation screen	Zygosity	Size in Mb of thegene- containinghomozygous region(rank)
*CDH3*	c.2200G>A	p.V734M	3*10^−5^	arRP	Druze	MOL1145	—	Homozygous	
*CDHR1*	c.1T>G	p.M1L	0	arRP	Druze	MOL1275	—	Homozygous	
*RPE65*	c.227A>C	p.H76P	0	arRP	Arab Muslim	MOL1026	—	Homozygous	
*RPE65*	c.329A>G	p.D110G	0	arLCA	Arab Muslim	MOL0093	—	Homozygous	35.7 (4)

A Sequence variant is considered as a possible pathogenic mutation if all of the following applies: co-segregation in the studied family, MAF < 0.001 by ExAC (http://exac.broadinstitute.org/), was not found in a homozygous state in the ExAC controls, and at least one of the programs predicted it to be a non-tolerated change.

**Table 3:** Pathogenic predictions of all novel missense mutations.

Gene	DNA change	Protein change	Mutation taster	PolyPhen	SIFT	MAF in various WES databases		
						Israeliin-housedatabase(N = 408)	EVS(N = 6,500)	ExAC(N = 66,000)
*ADAM9*	c.1087T>A	p.C363S	Disease causing	1	0.00 Damaging	0	0	0
*CDH3*	c.2200G>A	p.V734M	Disease causing	1	0.06 Tolerated	0	0	3*10^−5^
*CDHR1*	c.1T>G	p.M1L	Disease causing	0	0.54 Tolerated	0	0	0
*CNNM4*	c.1494C>A	p.D498E	Disease causing	1	0.00 Damaging	0	0	0
*RPE65*	c.227A>C	p.H76P	Disease causing	0.059	0.11 Tolerated	0	0	0
*RPE65*	c.329A>G	p.D110G	Disease causing	0.998	0.31 Tolerated	0	0	0
*USH2A*	c.6937G>T	p.G2313C	Disease causing	1	0.00 Damaging	1*10^−3^	8*10^−5^	1*10^−4^

**Table 4:** Clinical features of patients with identified disease causing mutations.

Patients ID (age, years)	Consanguinity	Diagnosis	Mutated Gene	Visual acuity	Refraction	Full Field ERG amplitudes (μV)		
						DA RodResponse(Avarage)	LA 30 Hz ConeFlicker (IT)	DA MixedRod-Cone(b, μV)
MOL0093 IV:2 (9)	2:2	arLCA	*RPE65*			ND	ND	ND
MOL0108 IV:2 (5)	No	adRP	*PFRP3*	0.4	+4.25		3.75 (43)	ND
MOL0108 III:7 (37)	No	adRP	*PFRP3*				ND	ND
MOL0108 III:13 (36)	No	adRP	*PFRP3*	3 m FC		ND	ND	ND
MOL011 II:2 (7)	No	arLCA	*AIPL1*	0.14 (15)	sph (15)	ND	ND	ND
MOL0113 II:3 (7)	No	arLCA	*AIPL1*	0.14 (15)	+2.75 (4)	ND	ND	ND
MOL0181 II:1 (26)	2:2	arRP	*RP1*	0.08	−6.25	ND	ND	ND
MOL0228 II:1 (16)	Distant	arRP	*FAM161A*	0.63	high myopia			
MOL0228 II:2 (12)	Distant	arRP	*FAM161A*	0.5	−3.50			
MOL0271 II:1 (15)	2:2	arRP	*PDE6A*	1.0 (8)		ND	ND	ND
MOL0271 II:2 (7)	2:2	arRP	*PDE6A*	1.0		Trace response	55 (38)	ND
MOL0425 II:2 (49)	No	arRP	*C2ORF71*	0.25	−10.25	ND	ND	ND
MOL0425 II:4 (47)	No	arRP	*C2ORF71*	HM		ND	ND	ND
MOL0589 II:2 (31)	2:1	arRP	*RDH12*	0.5 m FC		a-98 b-58	22 (41)	ND
MOL0635 II:2 (88)	2:2	ar late-onset RP	*PLA2G5*	HM		ND	ND	ND
MOL0745 III:3 (32)	2:2	arRP	*BBS1*	2 m FC	−7.50	Trace response	34 (45)	ND
MOL0745 IV:1 (23)	2:2	arRP	*BBS1*	HM	−5.50	Trace response	6 (44)	ND
MOL0816 III:6 (28)	No	arRP	*CYP4V2*	0.5		a-117 b-202	65 (38)	112
MOL0826 II:1 (37)	No	arRP	*MAK*	0.5	−1.75	ND (19)	ND (19)	ND (19)
MOL0835 II:1 (19)	2:2	arRD	*CDHR1*			ND	ND	ND
MOL0838 II:1 (38)	No	arRP	*ADAM9*	HM	0.9			
MOL0927 II:3 (55)	No	arRP	*CNGA1*	0.25				
MOL0990 III:2 (17)	Distant	arRP	*CNGB1*	0.10		a-12 b-40	19 (37)	ND
MOL1025 II:1 (17)	2:2	ar Fundus albipunctatus	*RDH5*	0.32		a-181 b-406	70 (29)	260
MOL1026 II:1 (21)	2:2	arRP	*RPE65*	1.0m FC	+4.5	ND	ND	ND
MOL1026 II:2 (7)	2:2	arRP	*RPE65*	0.4cm FC	+4.5	ND	ND	ND
MOL1109 II:2 (56)	2:2	arRP	*NRL*	HM		ND	ND	ND
MOL1125 II:1 (4)	No	arLCA	*TULP1*	0.25	+4.75	Trace response	Trace response	Trace response
MOL1145 III:3 (34)	No	arRP	*CDH3*	5cm HM		Trace response	Trace response	Trace response
MOL1145 III:4 (30)	No	arRP	*CDH3*	45 cm HM		Trace response	Trace response	Trace response
MOL1145 III:6 (33)	No	arRP	*CDH3*	35 cm HM		Trace response	Trace response	Trace response
MOL1234 II:2 (27)	2:2	arRP	*USH2A*	0.4 (24)		a-38 b-86	32 (40)	106
MOL1275 II:2 (28)	3:3	arRP	*CDHR1*	HM				
MOL1389 II:2 (44)	2:2	arRP	*RDH12*	HM		ND	ND	ND
CHRD1 II:1 (42)	3:3	arRP	*CNGB1*	0.63	−3.00	ND	ND	ND
CHRD2 II:2 (58)	3:3	arRP	*CNGB1*	0.5	−11.00			

ND- Not Detected, HM- hand motion, FC- finger counting, m- meter, cm- centimeter.
