# Supplementary material for: Whole Exome Sequencing Reveals Mutations in Known Retinal Disease Genes in 33 out of 68 Israeli Families with Inherited Retinopathies
Source: Sci Rep. 2015 Aug 26;5:13187. doi: 10.1038/srep13187 (PMC4549705; doi:10.1038/srep13187)

Supplementary Information

Whole Exome Sequencing Reveals Mutations in Known Retinal Disease Genes in 33 out of 68 Israeli and Palestinian Families with Inherited Retinopathies

Avigail Beryozkin[1], Elia Shevah[1], Adva Kimchi[1], Liliana Mizrahi-Meissonnier[1], Samer Khateb[1], Rinki Ratnapriya[2], Csilla H. Lazar[2,3], Anat Blumenfeld[1], Tamar Ben-Yosef[4], Yitzhak Hemo[1], Jacob Pe'er[1], Eduard Averbuch[1], Michal Sagi[5], Alexis Boleda[2], Linn Gieser[2], Abraham Zlotogorski[6], Tzipora Falik-Zaccai[7], Ola Alimi-Kasem[8], Samuel G. Jacobson[9], Itay Chowers[1], Anand Swaroop[2*], Eyal Banin[1*], Dror Sharon[1*]

Supplementary Table S1: A list of 226 known IRD genes that were analyzed in this study by WES.

ABCA4, ABCC6, ABHD12, ACBD5, ADAM9, ADAMTS18, AHI1, AIPL1, ALMS1, ARL2BP, ARL6, ARMS2, ATXN7, BBIP1, BBS1, BBS10, BBS12, BBS17, BBS2, BBS4, BBS5, BBS7, BBS9, BEST1, C1QTNF5, C2, C21orf2, C2orf71, C3, C8orf37, CA4, CABP4, CACNA1F, CACNA2D4, CAPN5, CC2D2A, CDH23, CDH3, CDHR1, CEP164, CEP250, CEP290, CERKL, CFB, CFH, CHM, CIB2, CLN3, CLRN1, CNGA1, CNGA3, CNGB1, CNGB3, CNNM4, COL11A1, COL2A1, COL9A1, CRB1, CRX, CSPP1, CYP4V2, DFNB31, DHDDS, DHX38, DMD, DTHD1, EFEMP1, ELOVL4, EMC1, ERCC6, EYS, FAM161A, FBLN5, FLVCR1, FSCN2, FZD4, GDF6, GNAT1, GNAT2, GNPTG, GPR125, GPR179, GPR98, GRK1, GRM6, GUCA1A, GUCA1B, GUCY2D, HARS, HK1, HMCN1, HMX1, HTRA1, IDH3B, IFT140, IFT172, IFT27, IMPDH1, IMPG1, IMPG2, INPP5E, INVS, IQCB1, ITM2B, JAG1, KCNJ13, KCNV2, KIAA1549, KIF11, KIZ, KLHL7, LAMA1, LCA5, LRAT, LRIT3, LRP5, LZTFL1, MAK, MERTK, MFRP, MKKS, MKS1, MTTP, MVK, MYO7A, NDP, NEK2, NEUROD1, NMNAT1, NPHP1, NPHP3, NPHP4, NR2E3, NRL, NYX, OAT, OFD1, OPN1LW, OPN1MW, OPN1SW, OTX2, PANK2, PAX2, PCDH15, PCYT1A, PDE6A, PDE6B, PDE6C, PDE6G, PDE6H, PDZD7, PEX1, PEX2, PEX7, PGK1, PHYH, PITPNM3, PLA2G5, PLK4, POC1B, PRCD, PROM1, PRPF3, PRPF31, PRPF4, PRPF6, PRPF8, PRPH2, PXMP3, RAB28, RAX2, RBP3, RBP4, RD3, RDH11, RDH12, RDH5, RGR, RGS9, RGS9BP, RHO, RIMS1, RLBP1, ROM1, RP1, RP1L1, RP2, RP9, RPE65, RPGR, RPGRIP1, RPGRIP1L, RS1, SAG, SDCCAG8, SEMA4A, SLC24A1, SLC7A14, SNRNP200, SPATA7, TEAD1, TIMP3, TLR3, TLR4, TMEM237, TOPORS, TREX1, TRIM32, TRPM1, TSPAN12, TTC8, TTLL5, TTPA, TUB, TULP1, UNC119, USH1C, USH1G, USH2A, VCAN, WDPCP, WDR19, WFS1, ZNF408, ZNF423, ZNF513.

Supplementary Table S2: Primers for all the suspected variants used in this study.

GENE NAME	FORWARD PRIMER	REVERSE PRIMER
ADAM9	GCAAAACATTTTCTTTGCGTTC	CATTTCCTGTTTTCCCAATG
AIPL1	ATGGGGTGAACTGAGTGAGC	AGTTTGCAGGACTGGCTTTG
BBS1	GGGTGTAGACATTGGGTTTCC	CCCTGGAGCCTCCCAAAC
CDHR1	CCTGATACAGACCAAGGACAACC	GGAGCAGAGAGCTTTCTGGGCTG
CNGA1	GCCTTTCCACTAATAAGACTTCTTTC	ATGCATGGGAAAATGTTTGG
CNGA1	AAGCTGGTCTGTTGGTGGAG	TTCCATGAGGTCATCTTTTGAG
CNGB1	TCCATGGAAGGGAGAGAC	CCATCCCTTGAGGAACTC
CNGB1	TGACCTCCCATAACCCTTCC	GAGTTGACTGGGAGCTGGTG
CNNM4	TAATGTTTTGGTTGAGGGAG	AGCTAGGGACACCAAGTTTC
CYP4V2	CTTGATCCACATGTTCTTCTTTG	AGAAACCCACCATCAAGTGG
C2ORF71	AAACAGGACCTCAGAGTCCAGCC	TTCTGGCCGAGCTGGGATTCATAA
FAM161A	TGAGCATGGTGGCACAAG	GCTGACCTACAAGGCAGAGG
MAK	AACGCTCCTTGAGAATGG	ACACCTGGCCTGTTAAGC
NRL	GACCTGGCGCTGACCCGGTTTCTGCATTCT	GCCACCCCCACCAGCCCCCACTACACCACA
NR2E3	GAGGGGAGCGTGCAGCCCTG	CACCCCTCCAGAACCCCTCAG
PDE6A	GGTGACTTTACTGCCAGCATC	CATACATATAATCATTGTTGCT
PRPF3	AAGTGACTTCAAAGACTGATTGTTG	CTCTTGATCCACACTAGGGTCA
PLA2G5	ATTAGGGATGGGGTCAGGAG	CAGGCTGGCTGTTCTCTAGG
RDH5	CAGATGCTCCCAGGAAGAAG	GAGTGGGCTGCTGTTAGTCC
RDH12	AATGCTCTGTCCCCCAGTC	CTCCCATACCCAATTTCCTG
RDH12	GCTAGGGGACTCCTTGCTAAC	AGGCCCCTGGACATTCTC
RPE65	TGCAAATGATGGAGAAAATG	TTTAGATGTGATTCAGATTGAGTGC
RP1	TTCAAGCCTAGGAGGTTGTTG	ATTGAAGCATGGATTTTGCC
SPATA7	AAAAACCAATTTTCTATTTTGTTCC	GCCAAATCTGACCCAATGTC

TSPAN12	TGACAGATATAGCTCTGGGTACAAA	GGAAAATTTCATTGGCATATTG
TULP1	AATCACAGAGCTCCCCAGAG	ACTGTGGTGGGTGCTCTACC
USH2A	AAGTATTGCTGGCAAGTGGC	GATCTTCACTACTACTGGTTTTGGG
USH2A	CACATCAAGAGTGCTTGCTTTC	CACCGCCACTTACTTCTTCC
CDH3	TGCTGTAGTCTCTTTGGCCC	GCTGGTTGGTGGTGGAGTC
CDHR1	TACCCCCATTGTGGTCTCT	CAAGCCTACAGCTCTGGTTC
RPE65	GGGCTGGAAATGAAAATCAC	GGCCCTACTTTGAGGAGGAG
RPE65	CCCTTTATTCTTCATGTTGTGC	AGAGGCAATCAGTGCAGTCC

Supplementary Table S3: A list of published Israeli families with nonsyndromic IRDs

Gene	Retinal phenotype	Number of Families	Inheritance pattern	Reference
ADAM9	CRD	2	AR	1
AIPL1	LCA	5	AR	2
ARL2BP	RP	1	AR	3
BBS1	RP	4	AR	4
CDHR1	CRD	1<br>1	AR	5<br>6
CEP290	LCA	1	AR	7
CERKL	RP / CRD	7	AR	8
CRB1	Early RP	1<br>1<br>15	AR	9<br>10<br>11
C2ORF71	RP	2<br>2	AR	12<br>13
C8ORF37	RP	2<br>1	AR	14<br>6
DHDDS	RP	15	AR	15
EYS	RP	10<br>1	AR	16<br>7
FAM161A	RP	20	AR	16
GUCY2D	LCA<br>CRD	4<br>6	AR<br>AD	2<br>6
IMPG2	RP	2	AR	17
LCA5	LCA	1	AR	7
MAK	RP	1	AR	18
MFRP	RP	1	AR	7

NR2E3	ESCS	10	AR	[19]
	GFS, RP	2		[7]
PDE6G	Early RP	1	AR	[20]
PDE6B	RP	1	AR	[21]
PRCD	RP	9	AR	[22]
PROM1	CRD	1	AR	[7]
		1		[23]
RAB28	CRD	1	AR	[24]
RDH5	AA	14	AR	[25]
RDH12	Early RP	7	AR	[7]
		1		[10]
RPE65	LCA/RP	10	AR	[2]
RPGR	RP	1	X-lined	[26]
TULP1	Early RP	1	AR	[7]
		2		[27]
USH1C	RP	9	AR	[28]
USH2A	RP	1	AR	[29]

The list includes phenotypes that were studied in the current study (mainly nonsyndromic RP, LCA, and CRD). Syndromic forms (mainly Usher syndrome and Bardet-Biedl syndrome) and maculopathies (mainly Stargardt disease and Best disease) were excluded from this table. Abbreviations: RP- Retinitis pigmentosa, CRD- Cone-rod degeneration, LCA- Leber congenital amaurosis, ESCS- Enhanced S-cone syndrome, GFS- Goldmann-Favre syndrome, AA- Albipunctata albescens.

References:

1 Parry, D. A. *et al.* Loss of the metalloprotease ADAM9 leads to cone-rod dystrophy in humans and retinal degeneration in mice. *American journal of human genetics* **84**, 683-691 (2009).

2 Banin, E. *et al.* Molecular anthropology meets genetic medicine to treat blindness in the North African Jewish population: human gene therapy initiated in Israel. *Human gene therapy* **21**, 1749-1757 (2010).

3 Davidson, A. E. *et al.* Mutations in ARL2BP, encoding ADP-ribosylation-factor-like 2 binding protein, cause autosomal-recessive retinitis pigmentosa. *American journal of human genetics* **93**, 321-329 (2013).

4 Shevach, E. *et al.* Missense mutations in the BBS2 gene can be associated with nonsyndromic retinitis pigmentosa. *JAMA Ophthalmol (in press)* (2014).

5 Cohen, B. *et al.* A novel splice site mutation of CDHR1 in a consanguineous Israeli Christian Arab family segregating autosomal recessive cone-rod dystrophy. *Molecular vision* **18**, 2915-2921 (2012).

6 Lazar, C. H. *et al.* Whole exome sequencing reveals GUCY2D as a major gene associated with cone and cone-rod dystrophy in Israel. *Invest Ophthalmol Vis Sci* (2014).

7 Beryozkin, A. *et al.* Identification of mutations causing inherited retinal degenerations in the israeli and palestinian populations using homozygosity mapping. *Investigative ophthalmology & visual science* **55**, 1149-1160 (2014).

8 Auslender, N. *et al.* A common founder mutation of CERKL underlies autosomal recessive retinal degeneration with early macular involvement

among Yemenite Jews. *Investigative ophthalmology & visual science* **48**, 5431-5438 (2007).

9 Gerber, S. *et al.* A novel mutation disrupting the cytoplasmic domain of CRB1 in a large consanguineous family of Palestinian origin affected with Leber congenital amaurosis. *Ophthalmic genetics* **23**, 225-235 (2002).

10 Benayoun, L. *et al.* Genetic heterogeneity in two consanguineous families segregating early onset retinal degeneration: the pitfalls of homozygosity mapping. *American journal of medical genetics. Part A* **149A**, 650-656 (2009).

11 Beryozkin, A. *et al.* Mutations in CRB1 are a Relatively Common Cause of Autosomal Recessive Early-Onset Retinal Degeneration in the Israeli and Palestinian Populations. *Invest Ophthalmol Vis Sci* (2013).

12 Collin, R. W. *et al.* Mutations in C2ORF71 cause autosomal-recessive retinitis pigmentosa. *American journal of human genetics* **86**, 783-788 (2010).

13 Khateb, S. *et al.* A homozygous nonsense CEP250 mutation combined with a heterozygous nonsense C2orf71 mutation is associated with atypical Usher syndrome. *J Med Genet* **51**, 460-469 (2014).

14 Estrada-Cuzcano, A. *et al.* Mutations in C8orf37, Encoding a Ciliary Protein, are Associated with Autosomal-Recessive Retinal Dystrophies with Early Macular Involvement. *American journal of human genetics* (2011).

15 Zelinger, L. *et al.* A missense mutation in DHDDS, encoding dehydrodolichyl diphosphate synthase, is associated with autosomal-

recessive retinitis pigmentosa in Ashkenazi Jews. *American journal of human genetics* **88**, 207-215 (2011).

16 Bandah-Rozenfeld, D. *et al.* Homozygosity mapping reveals null mutations in FAM161A as a cause of autosomal-recessive retinitis pigmentosa. *American journal of human genetics* **87**, 382-391 (2010).

17 Bandah-Rozenfeld, D. *et al.* Mutations in IMPG2, Encoding Interphotoreceptor Matrix Proteoglycan 2, Cause Autosomal-Recessive Retinitis Pigmentosa. *American journal of human genetics* **87**, 199-208 (2010).

18 Ozgul, R. K. *et al.* Exome Sequencing and cis-Regulatory Mapping Identify Mutations in MAK, a Gene Encoding a Regulator of Ciliary Length, as a Cause of Retinitis Pigmentosa. *American journal of human genetics* **89**, 253-264 (2011).

19 Bandah, D., Merin, S., Ashhab, M., Banin, E. & Sharon, D. The spectrum of retinal diseases caused by NR2E3 mutations in Israeli and Palestinian patients. *Archives of ophthalmology* **127**, 297-302 (2009).

20 Dvir, L. *et al.* Autosomal-recessive early-onset retinitis pigmentosa caused by a mutation in PDE6G, the gene encoding the gamma subunit of rod cGMP phosphodiesterase. *American journal of human genetics* **87**, 258-264 (2010).

21 Goldenberg-Cohen, N. *et al.* Genetic heterogeneity and consanguinity lead to a "double hit": homozygous mutations of MYO7A and PDE6B in a patient with retinitis pigmentosa. *Mol Vis* **19**, 1565-1571 (2013).

22 Nevet, M. J., Shalev, S. A., Zlotogora, J., Mazzawi, N. & Ben-Yosef, T. Identification of a prevalent founder mutation in an Israeli Muslim Arab village confirms the role of PRCD in the aetiology of retinitis pigmentosa in humans. *Journal of medical genetics* **47**, 533-537 (2010).

23 Pras, E. *et al.* Cone-rod dystrophy and a frameshift mutation in the PROM1 gene. *Molecular vision* **15**, 1709-1716 (2009).

24 Roosing, S. *et al.* Mutations in RAB28, encoding a farnesylated small GTPase, are associated with autosomal-recessive cone-rod dystrophy. *American journal of human genetics* **93**, 110-117 (2013).

25 Pras, E. *et al.* Fundus albipunctatus: novel mutations and phenotypic description of Israeli patients. *Molecular vision* **18**, 1712-1718 (2012).

26 Banin, E. *et al.* A non-ancestral missense mutation in families with either recessive or semi-dominant X-linked retinitis pigmentosa. *American Journal of Medical Genetics Part A* **143A**, 1150-1158 (2007).

27 Abbasi, A. H., Garzozi, H. J. & Ben-Yosef, T. A novel splice-site mutation of TULP1 underlies severe early-onset retinitis pigmentosa in a consanguineous Israeli Muslim Arab family. *Molecular vision* **14**, 675-682 (2008).

28 Khateb, S. *et al.* Exome sequencing identifies a founder frameshift mutation in an alternative exon of USH1C as the cause of autosomal recessive retinitis pigmentosa with late-onset hearing loss. *PloS one* **7**, e51566 (2012).

29 Kaiserman, N., Obolensky, A., Banin, E. & Sharon, D. Novel USH2A mutations in Israeli patients with retinitis pigmentosa and Usher syndrome type 2. *Arch Ophthalmol* **125**, 219-224 (2007).

Supplementary Figure S1: Pedigrees with identified disease-causing mutations

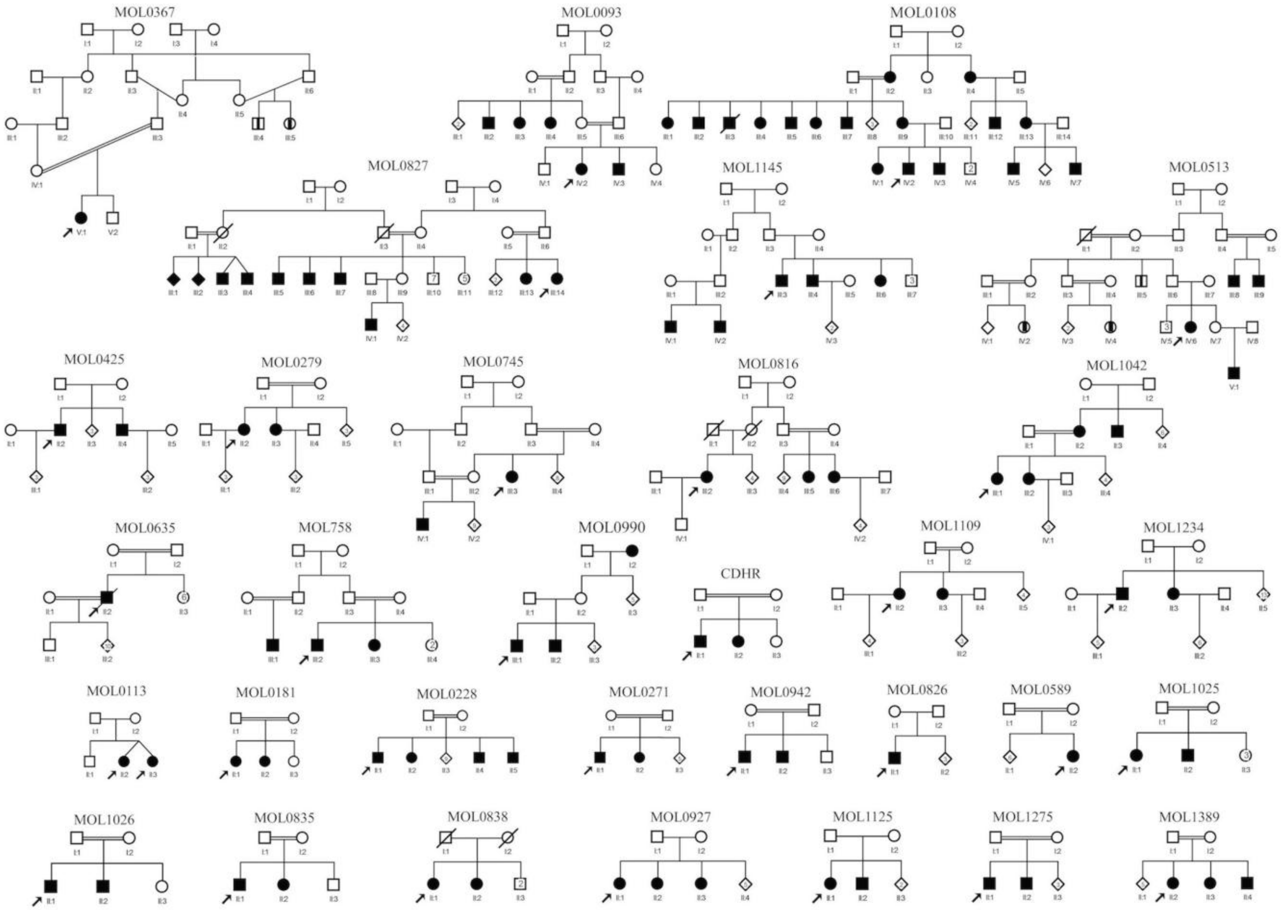

Supplement: Supplementary Information [file srep13187-s1.pdf]
